# Dual-energy CT based low flow rate, low dose CTPA and lung perfusion in the pulmonary diagnosis of post-COVID-19 syndrome

**DOI:** 10.3389/fmed.2026.1743139

**Published:** 2026-02-16

**Authors:** Zhi Jing, Lei Cui

**Affiliations:** 1 College of Medicine, Nantong University, Nantong, Jiangsu, China; 2 Department of Radiology, Nantong First People’s Hospital, Nantong, Jiangsu, China

**Keywords:** dual-energy CT, low flow rate and low dose, lung lesions, lung perfusion, post-COVID-19 syndrome

## Abstract

**Objective:**

To investigate the diagnostic value of low flow rate, low dose DECT in combination with D-dimer values and mMRC scores, in the assessment of lung lesions in patients with post-COVID-19 syndrome who have been infected with Omicron strains.

**Methods:**

A retrospective analysis was conducted on patients who underwent low-dose DECT pulmonary angiography (CTPA) between February 2023 and May 2024. Patients were categorized into positive or negative groups based on respiratory signs of post-COVID-19 syndrome, as determined by a respiratory physician, along with their corresponding mMRC scores. Demographic data (e.g., gender) were compared using the Chi-Square test. The Mann-Whitney *U* test was used for D-dimer, mean CT value, etc. The independent samples *t*-test was used for quantitative data (e.g., total lung volume). Inter-observer agreement for DECT parameters was assessed using the intraclass correlation coefficient (ICC). Diagnostic performance was evaluated using receiver operating characteristic (ROC) curves, with comparisons made via the Delong test.

**Results:**

A total of 92 cases were included. The positive group had a significantly higher median age and median D-dimer value. A higher mMRC score was associated with a greater likelihood of abnormal D-dimer values. No positive cases exceeded an mMRC grade of 3, and no severe clinical symptoms were observed. Inter-observer agreement for DECT parameters was excellent (ICC 0.8∼1.0). DECT perfusion revealed a significantly larger volume of perfused abnormal lung tissue and a lower mean iodine density in these areas in the positive group compared to the negative group (*P* < 0.001). The combined diagnostic model achieved a superior area under the curve (AUC) of 0.969, significantly outperforming individual parameters (*P* < 0.001).

**Conclusion:**

The low flow rate, low dose DECT enhances patient safety and accurately identifies functional lung abnormalities not easily detected by conventional CT or CTPA. When combined with clinical data such as mMRC scores and D-dimer levels, it significantly improves diagnostic accuracy for post-COVID-19 syndrome. This approach provides an objective basis for clinical diagnosis and can help guide appropriate treatment planning.

## 1 Introduction

Following the domestic Omicron pandemic in late 2022, some infected individuals experienced varying degrees of symptoms such as dyspnea, fatigue, palpitations, diminished sense of smell and taste, gastrointestinal dysfunction, headaches, mental fog, memory impairment, and difficulty concentrating within months after recovering from the acute phase (1, 2). Relevant literature indicates that during the initial COVID-19 pandemic, some patients with a history of SARS-CoV-2 infection also developed similar symptoms months after acute recovery. These symptoms have been termed post-COVID Syndrome, also known as “Long COVID,” by some experts and scholars (3, 4). The current clinical definition is “symptoms typically emerging within 3 months of SARS-CoV-2 infection, persisting for at least 2 months, with some patients experiencing symptoms lasting up to 6 months, and currently unexplained by alternative diagnoses” (5). The vast majority of these patients exhibit varying degrees of respiratory clinical manifestations. Beyond those who developed severe pneumonia during the acute phase of COVID-19, where pulmonary fibrosis contributes to symptoms, research indicates that microthrombi resulting from COVID-19-induced pulmonary vascular endothelial damage are also a primary cause of respiratory clinical symptoms (6, 7). Furthermore, clinical observations reveal that D-dimer levels in COVID-19 patients exhibit varying degrees of elevation during the progressive and recovery phases compared to healthy individuals, demonstrating high specificity and sensitivity (8–10).

Reviewing relevant literature from recent years reveals that the strains infecting these patients were predominantly the original Alpha variant and Delta variant, both significantly more virulent than the Omicron variant prevalent domestically since late 2022 (11–13). Furthermore, some researchers have employed dual-energy CT perfusion imaging to study the lungs of critically ill hospitalized patients and recovered discharged patients with prior α or Δ variant infections. These studies indicate that dual-energy CT perfusion can identify perfusion abnormalities not readily visible on plain chest CT scans from a functional imaging perspective (14, 15). Concurrently, researchers have compared lung SPECT/CT ventilation/perfusion (V/Q) testing (16) and hyperpolarized 129Xe pulmonary MRI imaging (17) with dual-energy CT lung perfusion. They concluded that the diagnostic efficacy of these two techniques is comparable to that of dual-energy CT lung perfusion. However, dual-energy CT lung perfusion offers advantages such as ease of operation, cost-effectiveness, non-invasiveness, shorter examination time, and lower radiation exposure to patients, making it highly valuable for clinical application.

At present, evidence remains limited regarding whether infection with the Omicron variant can similarly lead to post-COVID syndrome and how pulmonary involvement specifically manifests in this population. Accordingly, the present study aimed to evaluate the diagnostic utility of the modified Medical Research Council (mMRC) dyspnea scale for assessing pulmonary abnormalities in patients with post-COVID syndrome following Omicron infection. This evaluation was performed using a “one-stop” lung perfusion assessment enabled by the Lung Analysis module of a DECT post-processing system, based on low–flow-rate, low–dose DECT pulmonary angiography (CTPA), in combination with circulating D-dimer levels and mMRC scores. By integrating functional imaging with clinical indicators, this approach sought to provide objective evidence to support clinical diagnosis and to assist clinicians in therapeutic decision-making. Importantly, the reduced iodine contrast dose and injection rate further enhanced patient safety, reinforcing the clinical feasibility of this strategy.

## 2 Subjects and methods

### 2.1 Clinical data

All subjects in this section were derived from cases undergoing low-dose, low-flow CTPA examinations between February 2023 and May 2024. In addition to collecting general information such as age, gender, and medical history, D-dimer levels and mMRC scores were also recorded.

#### 2.1.1 Study subjects

The specific clinical criteria for diagnosing post-COVID-19 infection syndrome positivity were: (1) a history of Omicron variant infection; (2) recent symptoms including chest tightness, shortness of breath, or difficulty breathing, and cough; (3) for patients with Omicron variant infection history and positive physical signs, symptoms persisting for over 2 months and onset within 3 months post-infection.

**FIGURE 1:**
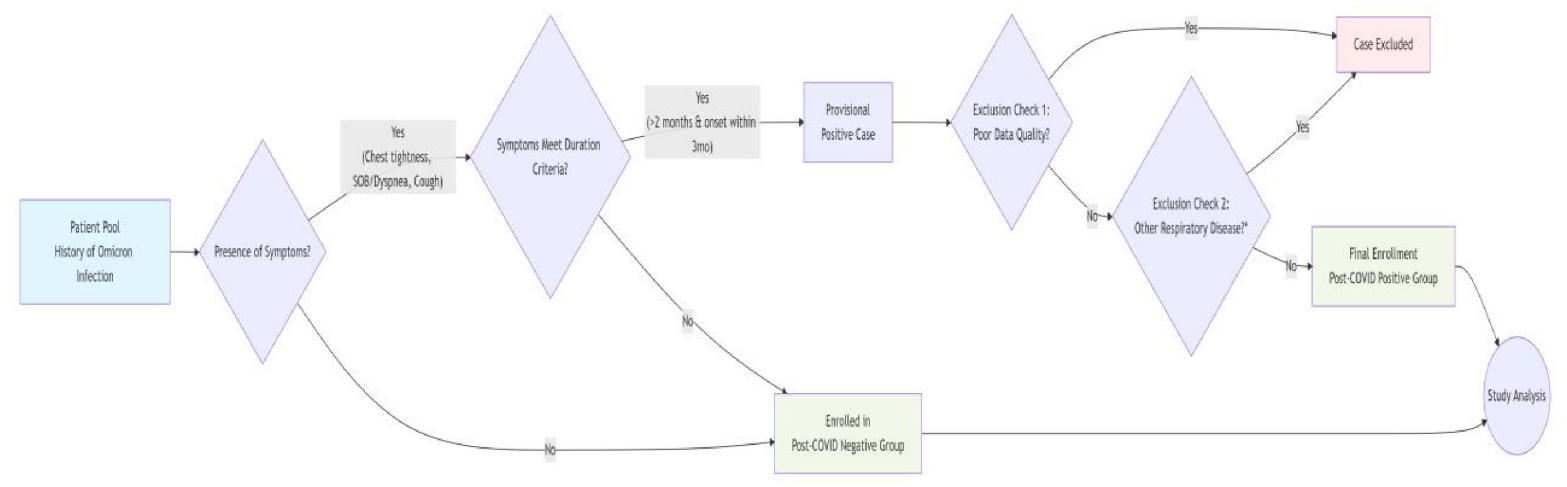
Inclusion and exclusion criteria.

All patients were diagnosed and grouped by respiratory physicians according to the above clinical criteria. Inclusion criteria: All patients meeting all criteria were enrolled in the positive group for study; patients with a history of Omicron infection but without positive physical signs were enrolled in the negative group for study. Exclusion criteria: (1) Cases with significant imaging artifacts or missing clinical/imaging data precluding diagnostic analysis; (2) Cases with a history or imaging findings suggestive of acute pulmonary embolism, emphysema, interstitial lung disease, tuberculosis, asthma, thoracic malignancy, or acute upper respiratory infection—conditions likely to significantly impact this study (see Figure 1 for details).

Based on the above inclusion and exclusion criteria, 17 non-compliant cases were excluded. A total of 92 compliant cases were enrolled: 42 in the positive group (25 males, 17 females) and 50 in the negative group (22 males, 28 females).

#### 2.1.2 mMRC score

The modified Medical Research Council scale (mMRC score) is used to quantify the severity of patients’ symptoms. The severity of respiratory symptoms such as dyspnea and shortness of breath in both groups is classified into grades 0–4:Grade 0 (no significant symptoms): No obvious clinical symptoms or only mild shortness of breath after strenuous physical activity; Grade 1 (mild symptoms): Shortness of breath or dyspnea during brisk walking on level ground or climbing gentle slopes; Grade 2 (moderate symptoms): Slower walking speed than age-matched asymptomatic individuals during brisk walking on level ground or climbing gentle slopes, requiring rest; Grade 3 (severe symptoms): Requiring rest to catch breath after walking 100 meters on level ground or walking for several minutes; Grade 4 (Severe Symptoms): Inability to leave home due to severe dyspnea or marked difficulty breathing when dressing/undressing.

#### 2.1.3 D-dimer levels

D-dimer, a key thrombotic biomarker, is widely used clinically for diagnosing and assessing prognosis in conditions such as deep vein thrombosis, microvascular thromboembolism, disseminated intravascular coagulation, and malignant tumors. Clinically, levels exceeding 500 μg/L are considered abnormal.

### 2.2 Examination method

Both patient groups underwent imaging on a Siemens third-generation dual-source CT scanner (Somatom Force, Siemens Healthcare) using a low-flow, low-dose CTPA dual-energy scanning protocol. Patients were positioned supine with arms raised, feet-first orientation, and breath-held exposure during quiet breathing. Specific scanning parameters: A tube voltage: 70 kV B tube voltage: 150 kV CARE Dose 4D automatic tube current modulation enabled Girdle rotation time: 0.25 s Pitch: 0.6 The ROI monitoring circle was positioned within the pulmonary artery trunk with a trigger threshold of 60 HU. Scanning commenced 2–3 s after automatic triggering. Reconstruction parameters included a slice thickness and slice spacing of 1.0 mm, with a D30f convolution kernel. All patients received 35 mL of contrast agent (350 mg/mL iopamidol) administered at a rate of 2.5 mL/s.

### 2.3 Image post-processing, identification and outlining of morphological and perfusion abnormalities in CT plain scans and CTPA

A technician concealed all personal information—including patient names, ages, genders, and image IDs—from the original images of both case groups. The original sequence of cases in each group was then randomly rearranged twice. Finally, the reordered original images of all cases were assigned to two experienced senior radiologists of comparable skill levels. Both physicians independently performed image post-processing, image review, delineation of perfusion abnormalities, and measurements simultaneously in separate environments.

#### 2.3.1 Image post-processing

Original images from included cases were imported into the Siemens post-processing workstation (syngo.via VB20A). All processing and reconstructions were based on 1 mm-thick 90 kVp chest plain scan images and original CTPA images. Standard procedures included multi-planar reformation (MPR), maximal intensity projection (MIP), and volume rendering (VR). Switch to Dual Energy mode, open the 1 mm slice thickness 90 kVp CTPA virtual reconstruction image, adjust to lung window, and perform iodine density mapping and effective atomic number (EAN) mapping in Lung Analysis for the Tra, Sag, and Cor planes. When reconstructing the EAN map, adjust the pseudocolor band window width so that the EAN values represented by the bands correspond to organ tissues in descending order: “red, orange, yellow, green, blue, purple, black.” Ensure uniform width for each color band within the pseudocolor scale.

#### 2.3.2 CT plain scan and CTPA morphology

Review the chest CT plain scan and CTPA sequences for the studied cases to further observe and diagnose:—The presence of abnormal structure in both lung parenchyma that do not significantly affect pulmonary ventilation or perfusion, and their nature (e.g., ground-glass opacities, solid nodular shadows, sparse fine fibrous strands, calcifications, etc.)—Whether the morphology and course of bronchial structures in each lung lobe and segment are normal and natural, and whether abnormal structure exist within the bronchi; In the CTPA sequences, observe whether embolism is present in the pulmonary artery trunk and its branches at all levels.

#### 2.3.3 Identification and segmentation of perfusion abnormalities

**FIGURE 2:**
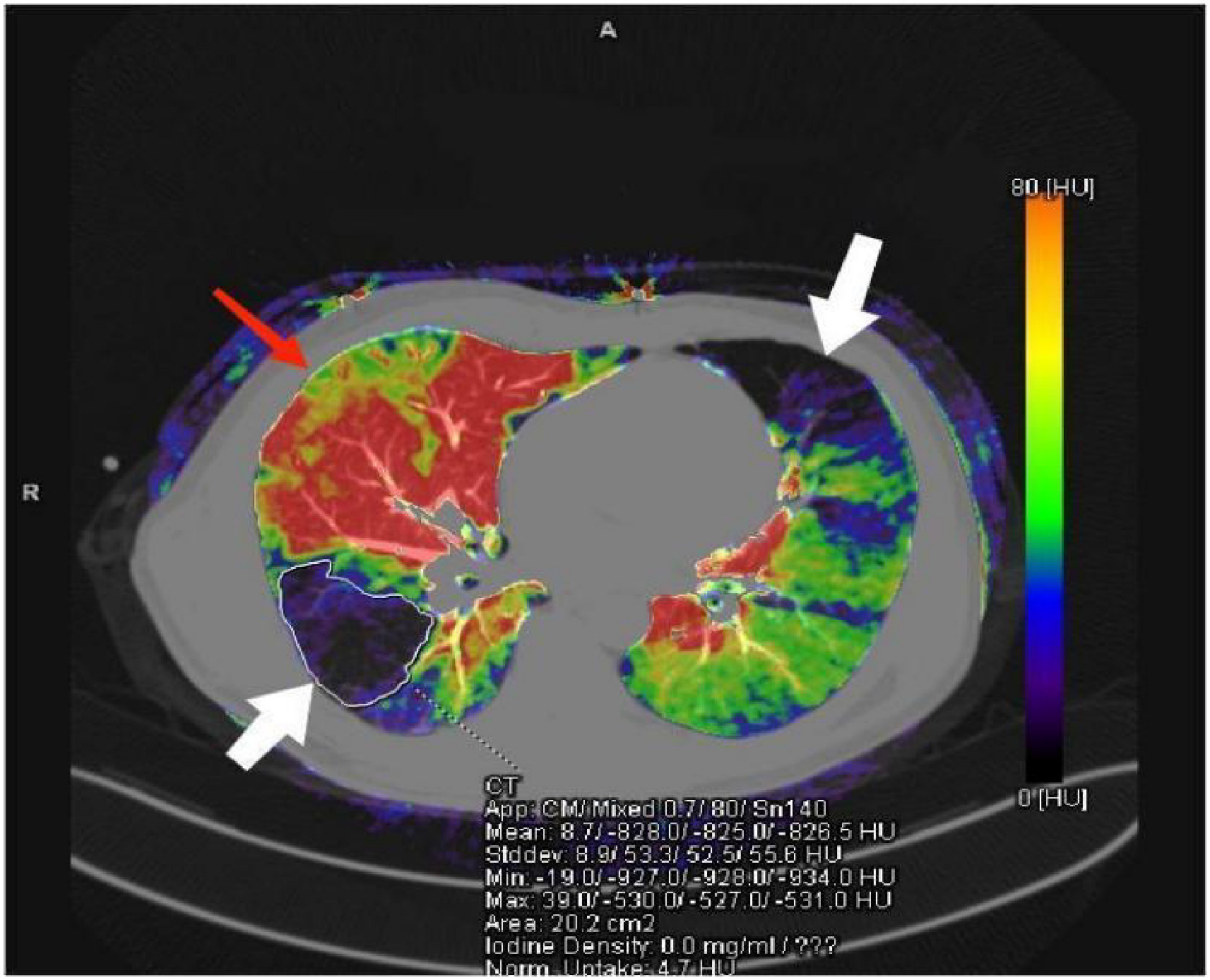
Schematic diagram of anomalous perfusion areas. The purple-black areas indicated by white arrows represent regions of reduced/deficient perfusion; the orange-red areas indicated by red arrows represent regions of enhanced perfusion.

Identify perfusion abnormalities on the effective atomic number map, categorizing regions as either perfused normally or abnormally. Abnormal perfusion areas include hypoperfusion or defect zones (corresponding to purple/black pseudo-color bands) and abnormal hyperperfusion zones (corresponding to red/orange pseudo-color bands). Segment abnormal perfusion regions layer-by-layer using the Freehand tool with 1 mm slice thickness, taking care to exclude tissues prone to false perfusion enhancement, such as the pleura, pulmonary artery branches above the pulmonary segment, mediastinum, and cardiac great vessels (as shown in Figure 2).

### 2.4 Measurement and calculation

#### 2.4.1 Measurement of morphological quantitative parameters in plain CT and CTPA

The morphological quantitative parameters measured in plain chest CT and CTPA primarily include the mean CT value, standard deviation of CT values, median CT value, interquartile range of CT values for both lungs in the two patient groups; as well as the mean CT value and standard deviation of CT values for the main pulmonary artery and its branches in both groups. Within the post-processing system’s reconstruction module, select the lung-window CT scan and CTPA cross-sectional sequences from the raw images. Using a circular Region of Interest (ROI) tool, delineate several areas of equal size within the lung parenchyma of both lungs. During delineation, carefully avoid major vessels in the lungs and mediastinum, as well as any abnormal structure within the lung parenchyma. Calculate the mean CT value for the lung parenchyma by averaging multiple measurements. Then, use the circular ROI to delineate the center of the pulmonary artery trunk and its branches at each level. Use the measured values as the mean CT values and standard deviations for the pulmonary artery trunk and its branches at each level in this case. Finally, using SPSS 26.0 software, further calculate the mean CT values, standard deviation of CT values, median CT values, and interquartile ranges for both groups of patients’ lung parenchyma, as well as the median CT values and interquartile ranges for the main pulmonary artery and its branches in both groups.

#### 2.4.2 Measurement and calculation of absolute volume of perfusion abnormal regions

Perfusion abnormal regions were categorized into purple-black hypoperfusion or defect areas and red-orange hyperenhancement areas. After delineating the edges of abnormal regions layer by layer using the Freehand tool with 1 mm slice thickness, clicking the “Extend ROI Text” option in the top list displayed all measurements around the delineated region. The “Area” value represented the delineated region’s surface area. Let the absolute volume (surface area) of the perfusion abnormality region be Vnu (Snu), the volume (surface area) of the perfusion reduction or defect region be Vre/dam (Sre/dam), the volume (surface area) of the perfusion abnormality enhancement region be Vstr (Sstr), and the slice thickness be h. The specific calculation formulas are as follows:


$$\sum V_{u\,n\,u}=\sum V_{r\,e/d\,a\,m}+\sum V_{s\,t\,r}$$

$$\sum V_{r\,e/d\,a\,m}=\overset{n}{\underset{i=1}{\sum }}S_{r\,e/d\,a\,m_{i}}\cdot h\left(n\geq i,n,i\in N^{{}^{*}}\right)$$

$$\begin{array}{c} \sum V_{s\,t\,r}=\overset{n}{\underset{j=1}{\sum }}S_{s\,t\,r_{j}}\cdot h\left(n\geq j,n,j\in N^{{}^{*}}\right) \end{array}$$

#### 2.4.3 Measurement of total lung volume and calculation of absolute volume of normal lung perfusion areas

In the post-processing system, enter Volume mode and select CT_LungCare. Use the Freehand method to delineate bilateral lung tissue, taking care to exclude organs and structures such as the pleura, mediastinum, and major cardiac vessels. After delineation, locate Volume in the lower section of the post-processing page to obtain the total lung volume. Let Vtot denote the total lung volume and Vnor denote the absolute volume of the normally perfused lung region. The specific calculation formula is as follows:


$$V_{n\,o\,r}=V_{t\,o\,t}-\sum V_{u\,n\,u}=V_{t\,o\,t}-\left(\sum V_{r\,e/d\,a\,m}+\sum V_{s\,t\,r}\right)$$

#### 2.4.4 Calculation of relative volume percentages for abnormal perfusion areas and normal lung perfusion areas

Let the relative volume percentage of areas with reduced or absent perfusion be V%re/dam, the relative volume percentage of areas with abnormal perfusion enhancement be V%str, and the relative volume percentage of normal lung perfusion areas be V%nor. The specific calculation formula is as follows:


$${V\%}_{r\,e/d\,a\,m}=\frac{\sum V_{r\,e/d\,a\,m}}{V_{t\,o\,t}}\cdot 100\%$$

$${V\%}_{s\,t\,r}=\frac{\sum V_{s\,t\,r}}{V_{t\,o\,t}}\cdot 100\%$$

$${V\%}_{n\,o\,r}=\frac{V_{n\,o\,r}}{V_{t\,o\,t}}\cdot 100\%=\frac{V_{t\,o\,t}-\left(\sum V_{r\,e/d\,a\,m}+\sum V_{s\,t\,r}\right)}{V_{t\,o\,t}}\cdot 100\%$$

#### 2.4.5 Measurement and calculation of average iodine density in normal lung perfusion areas and measurement of abnormal perfusion areas

After delineating the abnormal area, the measured average iodine density for that region can be found in the Iodine Density section. To measure iodine density in normal lung perfusion areas, simply delineate three or more equal-sized normal perfusion ROIs arbitrarily and take the average iodine density. denoted as nor. The iodine densities for the remaining areas with reduced or absent perfusion (re/dam) and those with abnormal enhanced perfusion (str) are separately defined. The specific calculation formulas are as follows:


$${\bar{D}}_{r\,e/d\,a\,m}=\frac{\overset{n}{\underset{i=1}{\sum }}S_{r\,e/d\,a\,m_{i}}\,D_{r\,e/d\,a\,m_{i}}\cdot h}{\sum V_{r\,e/d\,a\,m}}\left(n\geq i,n,i\in N^{{}^{*}}\right)$$

$$\begin{array}{c} {\bar{D}}_{s\,t\,r}=\frac{\overset{n}{\underset{j=1}{\sum }}S_{s\,t\,r_{j}}\,D_{s\,t\,r_{j}}\cdot h}{\sum V_{s\,t\,r}}\left(n\geq j,n,j\in N^{{}^{*}}\right) \end{array}$$

### 2.5 Statistical analysis

All qualitative and quantitative data were analyzed using SPSS 26.0 software. Qualitative variables such as gender were compared between groups using the chi-square test. Differences in mMRC classification grades and D-dimer levels were assessed using Fisher’s exact test to determine whether abnormalities existed between groups. Continuous quantitative data first underwent Shapiro-Wilk normality testing. Since total lung volumes in both groups followed a normal distribution, variables were expressed as mean ± standard deviation. Based on homogeneity of variance results, either independent samples *t*-tests or Student’s *t*-tests were used to compare quantitative differences between groups. Age, D-dimer levels, mean CT values and standard deviations of normal lung parenchyma in both groups, mean CT values and standard deviations of the pulmonary artery trunk and its branches, and other dual-energy CT perfusion parameters did not follow normal distribution. These variables were expressed as median and interquartile range (M[Q1,Q3]) and analyzed using the Mann-Whitney U test. The repeatability of dual-energy CT perfusion parameters measured by two physicians was assessed using the ICC (Intraclass Correlation Coefficient) test. An ICC coefficient between 0.8 and 1.0 indicates very strong agreement; between 0.6 and 0.8 indicates strong agreement; ICC coefficients between 0.4 and 0.6 indicate moderate agreement; between 0.2 and 0.4 indicate poor agreement; and below 0.2 indicate very poor agreement. Diagnostic performance of each feature indicator was analyzed using ROC curves, evaluating AUC values, sensitivity, and specificity. AUC values > 0.9 indicate excellent diagnostic performance; An AUC value between 0.8 and 0.9 indicates good diagnostic performance; an AUC value between 0.6 and 0.8 indicates fair diagnostic performance; an AUC value < 0.6 indicates that the indicator has a certain degree of randomness and poor diagnostic performance. Comparisons between different ROC curves were performed using the DeLong test, with *P* < 0.05 (two-tailed) indicating statistically significant differences.

## 3 Results

### 3.1 Clinical data analysis

#### 3.1.1 General data analysis

The gender distribution of participants was comparable between groups: the positive group comprised 25 males and 17 females, whereas the negative group included 22 males and 28 females. Chi-square analysis revealed no statistically significant difference in sex distribution between groups (χ^2^ = 2.201, *P* = 0.138).

Both age and D-dimer levels exhibited non-normal distributions in both groups (Shapiro–Wilk test, *P* < 0.05) and are therefore presented as median (IQR). Median age was significantly higher in the positive group at 74.5 years (IQR 65.3–80.0) compared to 55.0 years (IQR 44.8–63.0) in the negative group (*Z* = –5.909, *P* < 0.001). Similarly, median D-dimer levels were markedly elevated in the positive group [533.0 μg/L (IQR 490.8–703.3)] relative to the negative group [269.0 μg/L (IQR 222.3–309.0)] (*Z* = –8.031, *P* < 0.001). Detailed values are presented in Table 1.

**TABLE 1:** Age, D-dimer levels, and corresponding *Z*-values and *p*-values for the two patient groups.

Project	Positive group	Negative group	*Z*	*P*
Age	74.5 (65.3, 80.0)	55.0 (44.8, 63.0)	−5.909	<0.001
D-dimer (μg/L)	533.0 (490.8, 703.3)	269.0 (222.3, 309.0)	−8.031	<0.001

#### 3.1.2 Analysis of D-dimer levels and mMRC scores

Among the 92 cases included in this study, all 50 asymptomatic cases in the negative group showed D-dimer levels below 500 μg/L and were classified as mMRC grade 0.

Within the positive group, the distribution of mMRC scrores and corresponding D-dimer status was as follows: 11 patients were classified as mMRC grade 1, of whom two exhibited abnormal D-dimer levels and nine had normal levels; 21 patients were classified as grade 2, with 17 showing abnormal D-dimer levels and four within the normal range; 10 patients were classified as grade 3, all demonstrating abnormal D-dimer levels. No patients were assigned to grade 4.

Analysis of D-dimer abnormalities stratified by mMRC scores revealed that among grade 1 cases, 81.8% had normal D-dimer levels and 18.2% were abnormal. In grade 2 cases, 19.0% had normal D-dimer levels, whereas 81.0% exhibited abnormal levels. All grade 3 cases (*n* = 10) demonstrated abnormal D-dimer values (100%).

Fisher’s exact test indicated a statistically significant association between D-dimer status and mMRC severity grade (*P* < 0.001). *Post-hoc* comparisons showed that the difference between grade 1 and the other two grades was statistically significant (*P* < 0.001), whereas no significant difference was observed between grades 2 and 3 (*P* = 0.642). Detailed data are presented in Table 2.

**TABLE 2:** Cross-tabulation of D-dimer levels and mMRC scores in the positive group.

Project			mMRC scores			Total
			Grade1	Grade2	Grade3	
Is the D-dimer level normal?	Y	Number	9^a^	4^b^	0^b^	13
		Percentage	81.8%	19.0%	0.0%	31.0%
	N	Number	2^a^	17^b^	10^b^	29
		Percentage	18.2%	81.0%	100.0%	69.0%
Total		Number	11	21	10	42
		Percentage	100.0%	100.0%	100.0%	100.0%

Both ^a^ and ^b^ indicate subsets of the mMRC classification categories, and the column proportions for these categories do not differ significantly from one another.

### 3.2 Reproducibility of measurements by two diagnostic physicians

The reproducibility of dual-energy CT perfusion parameters measured by two diagnosticians across both case groups was assessed using the ICC (Intraclass Correlation Coefficient) test. As shown in the table, the ICC coefficients for the two diagnosticians’ measurements of reduced perfusion/damaged volume (Vre/dam), enhanced perfusion volume (Vstr), and normal perfusion volume (Vnor) across both case groups ranged from 0.8 to 1.0, indicating excellent consistency. Specifically, in the positive group:- Volume of reduced/deficient perfusion: 0.945 (95% CI: 0.899–0.970) —Volume of enhanced perfusion: 0.957 (95% CI: 0.921–0.977) —Volume of normal perfusion: 0.993 (95% CI: 0.987–0.996) In the negative group, the volume with reduced/deficient perfusion was 0.821 (95% CI: 0.705–0.894), the volume with enhanced perfusion was 0.873 (95% CI: 0.787–0.926), and the volume with normal perfusion was 0.998 (95% CI: 0.997–0.999). All differences were statistically significant (*P* < 0.001) (see Table 3 for details).

**TABLE 3:** Inter-observer agreement among two diagnostic physicians.

Project	Positive group			Negative group		
	*V*_*re/dam*_	*V*_*str*_	*V*_*nor*,_	*V*_*re/dam*_	*V*_*str*_	*V*_*nor*,_
ICC	0.945	0.957	0.993	0.821	0.873	0.998
95%CI	0.899 ∼ 0.970	0.921 ∼ 0.977	0.987 ∼ 0.996	0.705 ∼ 0.894	0.787 ∼ 0.926	0.997 ∼ 0.999
*P*	<0.001	<0.001	<0.001	<0.001	<0.001	<0.001

Relative volume percentages and regional average iodine density are calculated values based on the aforementioned measurement data and therefore are not included in the consistency study.

### 3.3 Qualitative analysis of morphology in non-contrast CT, CTPA, and dual-energy CT perfusion

**FIGURE 3:**
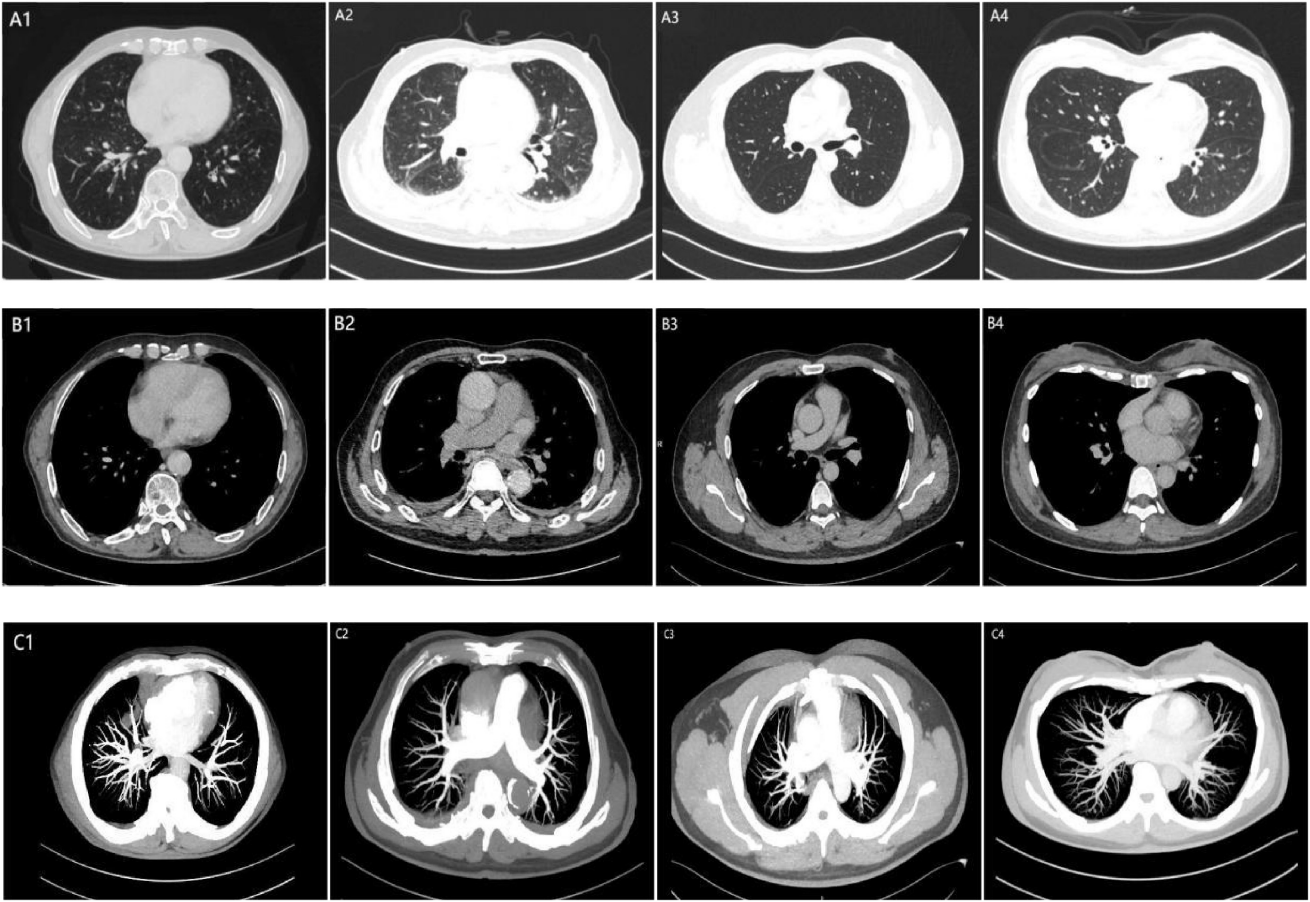
Chest CT plain scan and CTPA of two case groups. 1 (Male, 78 years old), 2 (Male, 85 years old) represent the positive group; 3 (Male, 33 years old), 4 (Female, 36 years old) represent the negative group; A, Plain scan lung window; B, Plain scan mediastinal window; C, CTPA (Tra-MIP).

Qualitative analysis of chest plain CT and CTPA revealed abnormal structure in both lung parenchyma in 16 cases: 6 in the positive group and 10 in the negative group. The remaining 76 cases demonstrated no other overt structural abnormalities were identified on plain CT, including 36 in the positive group and 40 in the negative group. The difference in the presence or absence of abnormal structure in both lung parenchyma between the two groups showed no significant statistical difference (χ^2^ = 0.519, *P* = 0.471). Additionally, no significant embolism was observed in the pulmonary artery and its branches in either group, with good filling. For example, as shown in Figure 3, in the plain chest CT scans (A, B) of both groups, except for a small amount of right pleural effusion and aortic sclerosis visible in the image of Case 2 in the positive group, no significant abnormal structure were observed in the lung parenchyma or mediastinum at the corresponding levels in the images of the other cases in both groups. In the CTPA (C) of both groups, no significant embolism was seen in the main pulmonary artery or its branches.

**FIGURE 4:**
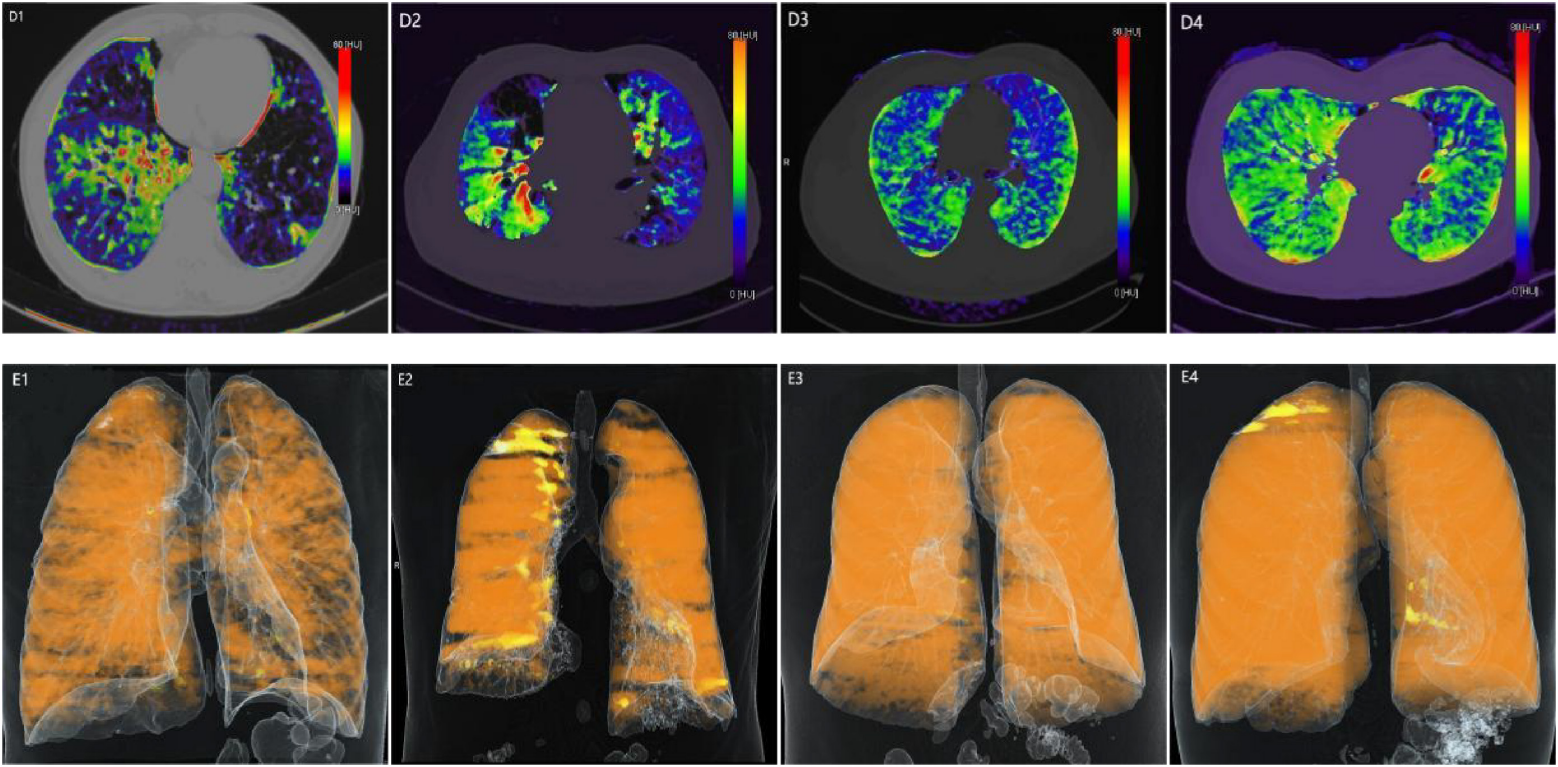
CT lung perfusion effective atomic number maps and VR maps for two case groups. D: Lung perfusion effective atomic number map E: Lung perfusion VR map.

Significant differences were observed between the two groups in the effective atomic number (EA) maps and VR maps of dual-energy CT lung perfusion. Specifically, patients in the positive group exhibited a higher prevalence of abnormal pulmonary perfusion compared to those in the negative group. For example, in Figure 4, images D and E (Cases 1 and 2) show areas of purple-black perfusion reduction/defects of varying degrees, as well as adjacent orange-red areas of abnormal perfusion enhancement. The VR images also reveal varying degrees of perfusion defects. In Cases 3 and 4, overall perfusion in both lungs was uniform and good, with no significant areas of abnormal perfusion.

### 3.4 Quantitative analysis of CT plain scan, CTPA, and dual-energy CT perfusion parameters

#### 3.4.1 Quantitative analysis of CT plain scan and CTPA parameters

The mean CT values and standard deviations of normal lung parenchyma in both groups, as well as those of the pulmonary artery trunk and its branches, exhibited skewed distributions. No statistically significant differences were observed between the two groups in the mean CT values and standard deviations of normal lung parenchyma or pulmonary artery structures (*P* > 0.05) (see Table 4 for details).

**TABLE 4:** Mean CT values, standard deviations, *Z*-values, and *P*-values for normal lung parenchyma, pulmonary artery trunk, and its branches in both groups.

Project	Positive group	Negative group	*Z*	*P*
Average CT value of normal lung parenchyma (Hu)	−708.50 (−808.25,−647.50)	−696.00 (−796.00, −605.00)	−0.874	0.382
SD (Hu)	8.89 (6.32, 11.89)	8.31 (6.08, 11.27)	−0.235	0.814
Average CT value of pulmonary arteries trunk(Hu)	316.26 (291.78, 348.36)	321.07 (283.72, 366.30)	−0.110	0.913
SD(Hu)	16.52 (13.02, 19.09)	17.03 (13.09, 19.97)	−0.329	0.742
Average CT value of upper right branch(Hu)	327.14 (301.18, 353.14)	309.56 (268.88, 358.53)	−1.564	0.118
SD(Hu)	17.01 (13.92, 20.43)	17.01 (14.66, 20.79)	−0.086	0.931
Average CT value of lower right branch(Hu)	321.85 (297.28, 349.35)	303.22 (260.99, 352.48)	−1.599	0.110
SD(Hu)	17.44 (14.92, 19.96)	17.86 (14.15, 19.72)	−0.274	0.784
Average CT value of upper left branch(Hu)	305.80 (284.50, 336.63)	314.35 (273.62, 358.00)	−0.118	0.906
SD(Hu)	17.34 (14.58, 20.11)	17.09 (14.16, 19.85)	−0.282	0.778
Average CT value of lower left branch(Hu)	299.61 (277.34, 330.80)	309.16 (268.07, 352.23)	−0.149	0.882
SD(Hu)	16.12 (13.01, 19.43)	15.50 (12.50, 20.0)	−0.363	0.717

42 cases in the positive group and 50 cases in the negative group.

#### 3.4.2 Quantitative analysis of dual-energy CT perfusion parameters

Combining the measurements and calculations from two diagnostic physicians, and reanalyzing the post-processed images, the average values from both physicians’ measurements and calculations were taken to derive the final measurement and calculation data. Except for total lung volume (bilateral), which followed a normal distribution, all other data points in both case groups exhibited skewed distributions. Student’s *t*-tests and Mann-Whitney U tests were performed on these data respectively. The results showed that, except for total lung volume (*P* = 0.447) and mean iodine density in perfused normal lung parenchyma (*P* = 0.111), which did not exhibit statistical differences, all other data points demonstrated statistically significant differences. Details are presented in Table 5.

**TABLE 5:** Dual-energy CT perfusion data, *Z/F* values, and *P*-values.

Project	Positive group	Negative group	*Z/F*	*P*
*V*_*tot*_ (cm^3^)	3140.9 263.1	3179.7 225.1	1.971	0.447
*V_*nor*_*,(cm^3^)	2920.7 (2736.1, 3104.4)	3061.2 (2888.8, 3286.6)	−3.386	0.001
*V%_*nor*_*	92.4 (90.7,95.5)	97.1 (96.8, 97.7)	−7.674	<0.001
*V*_*re/dam*_ (cm^3^)	144.9 (96.5, 197.0)	51.8 (32.8, 59.6)	−7.980	<0.001
*V%_*re/dam*_*	4.6 (3.2, 6.3)	1.7 (1.0, 1.8)	−8.003	<0.001
*V*_*str*_ (cm^3^)	88.4 (56.0, 109.3)	39.8 (35.5, 43.8)	−5.715	<0.001
*V%_*str*_*	2.9 (1.8, 3.4)	1.3 (1.1, 1.4)	−5.844	<0.001
$\bar{D}$*_*nor*_*(mg/mL)	1.4 (1.2, 1.5)	1.4 (1.3, 1.5)	−1.593	0.111
$\bar{D}$*_*re/dam*_*(mg/mL)	0.2 (0.1, 0.3)	0.3 (0.2, 0.5)	−4.016	<0.001
$\bar{D}$*_*str*_*(mg/mL)	2.3 (2.2, 2.5)	2.6 (2.5, 2.7)	−4.435	<0.001

42 cases in the positive group and 50 cases in the negative group.

### 3.5 ROC curve analysis of diagnostic performance for perfusion parameters

#### 3.5.1 ROC curves for volume parameters

The ROC curves for lung perfusion volume parameters were analyzed as follows: absolute volume of hypoperfused/deficient lung parenchyma (A1), absolute volume of hyperperfused lung parenchyma (A2), absolute volume of normally perfused lung parenchyma (A3), combined hypoperfusion–hyperperfusion diagnostic volume (A4), and the triad volume-based diagnostic combination incorporating all three parameters (A5), namely, absolute volumes of hypoperfused, hyperperfused, and normally perfused lung parenchyma.

**FIGURE 5:**
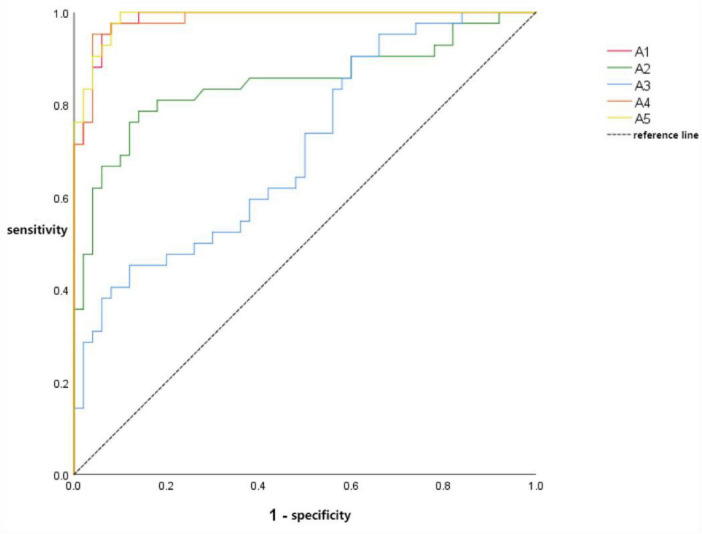
A1-A5 ROC curve.

Graphical analysis and tabulated results revealed the following diagnostic performance: A1:AUC = 0.905 (95% CI: 0.967–0.998), sensitivity = 97.6%, specificity = 92.0%; A2: AUC = 0.847 (95% CI: 0.760–0.934), sensitivity = 78.6%, specificity = 86.0%; A3: AUC = 0.706 (95% CI: 0.600–0.811), sensitivity = 45.2%, specificity = 88.0%; A4: AUC = 0.924 (95% CI: 0.965–0.998), sensitivity = 95.2%, specificity = 96.0%; A5: AUC = 0.928 (95% CI: 0.973–0.999), sensitivity = 97.6%, specificity = 92.0%. These results indicated that combined dual-parameter (A4) and triad (A5) models substantially improved diagnostic accuracy compared with individual volume parameters. Representative ROC curves are shown in Figure 5, and detailed values are summarized in Table 6.

**TABLE 6:** Differences in AUC values of A1-A5 ROC curves.

Project	AUC value difference	95%CI	*Z*	*P*
A1-A2	0.138	0.055∼0.221	3.248	0.001
A1-A3	0.279	0.171∼0.387	5.057	<0.001
A1-A4	0.001	−0.005∼0.007	0.299	0.765
A1-A5	−0.003	−0.010∼0.003	−0.962	0.336
A2-A3	0.141	0.009∼0.274	2.093	0.036
A2-A4	−0.137	−0.222 to −0.051	−3.135	0.002
A2-A5	−0.141	−0.225 to −0.056	−3.268	0.001
A3-A4	0.278	0.168∼0.388	4.969	<0.001
A3-A5	−0.282	−0.388 to −0.177	−5.241	<0.001
A4-A5	0.004	−0.005∼0.014	0.897	0.370

#### 3.5.2 Diagnostic performance ROC curves for iodine density parameters

**FIGURE 6:**
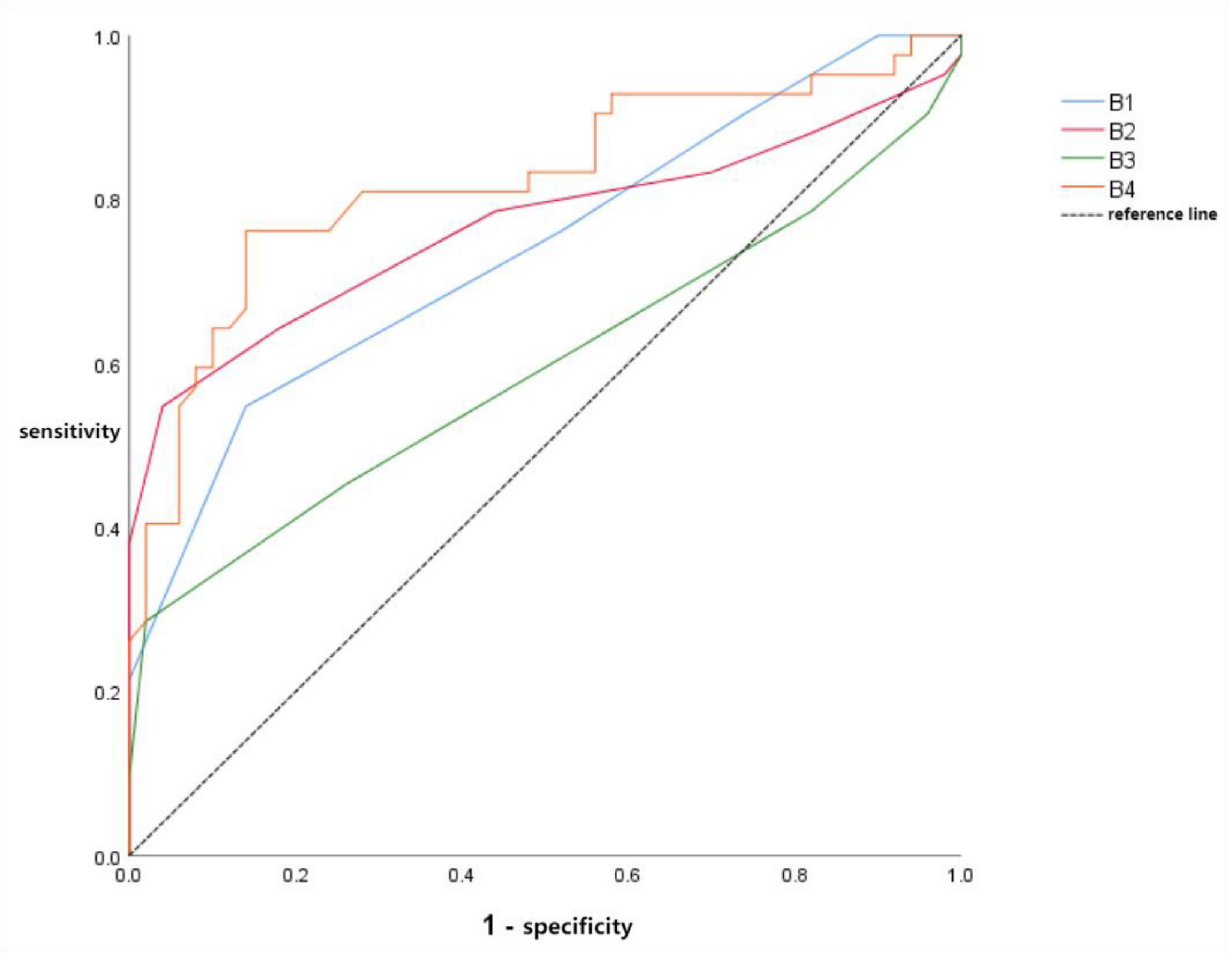
B1-B4 ROC curve.

The iodine density ROC curves are as follows: ROC curve for mean iodine density in lung parenchyma with reduced perfusion/lesions (B1), ROC curve for mean iodine density in lung parenchyma with enhanced perfusion (B2), the ROC curve for the mean iodine density of lung parenchyma in areas with normal perfusion (B3), and the triple-parameter ROC curve (B4) combining the mean iodine densities of areas with reduced/deficient perfusion, areas with enhanced perfusion, and areas with normal perfusion. Graphical analysis and table consultation revealed that B1 had an AUC of 0.738 (95% CI: 0.635–0.841), sensitivity of 54.8%, and specificity of 86.0%. B2 exhibited an AUC of 0.767 (95% CI: 0.660–0.873), sensitivity of 54.8%, and specificity of 96.0%. B3 had an AUC of 0.595 (95% CI: 0.473–0.717), with a sensitivity of 28.6% and specificity of 98.0%. The AUC value for B4 was 0.823 (95% CI: 0.732–0.913), with a sensitivity of 76.2% and specificity of 86.0% (see Figure 6 and Table 7 for details).

**TABLE 7:** Differences in AUC values of B1-B4 ROC curves.

Project	AUC value difference	95%CI	*Z*	*P*
B1-B2	−0.029	−0.151∼0.093	−0.468	0.640
B1-B3	−0.143	−0.281 to −0.004	−2.022	0.043
B1-B4	−0.085	−0.162 to −0.008	−2.165	0.030
B2-B3	−0.172	−0.269 to −0.075	−3.484	<0.001
B2-B4	−0.056	−0.125∼0.014	−1.573	0.116
B3-B4	−0.228	−0.349 to −0.106	−3.669	<0.001

#### 3.5.3 ROC curve for multi-parameter combined diagnosis

**FIGURE 7:**
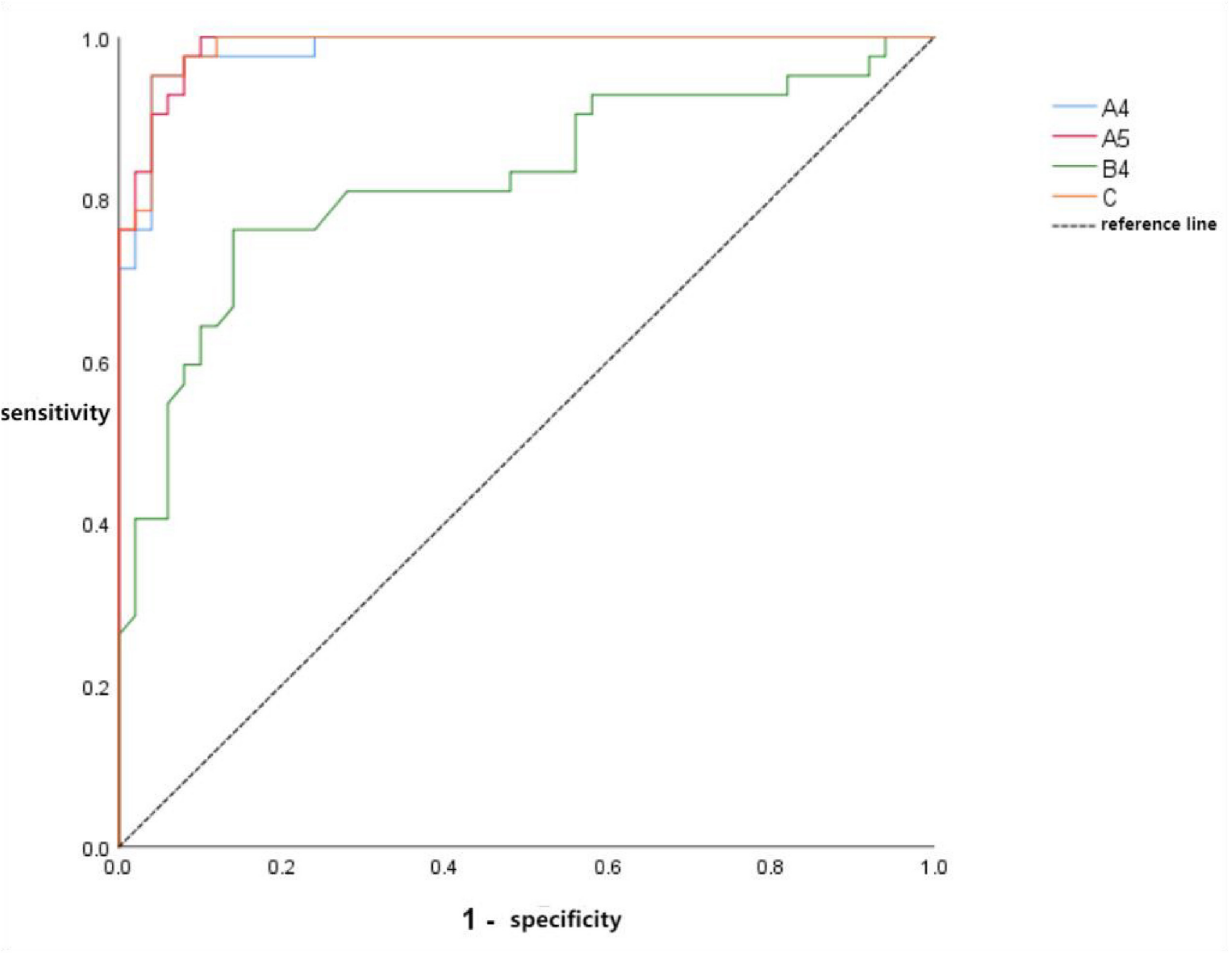
Combined diagnostic ROC curve.

Combining the volume ROC curve, iodine density ROC curve, and D-dimer value yielded a combined diagnostic ROC curve (C) with an AUC of 0.969 (95% CI: 0.976–0.999), sensitivity of 95.2%, and specificity of 96.0%. After pairwise comparisons using DeLong’s test among the following four ROC curves: reduced perfusion/perfusion defect-enhanced perfusion dual diagnostic (A4), triple volume diagnostic (A5), triple iodine density diagnostic (B4), and combined diagnostic (C), no statistically significant differences were found in AUC values. All three curves showed significantly higher AUC values than the dual diagnostic curve. defect-perfusion enhancement dual diagnostic ROC curve, the triple volume diagnostic ROC curve, and the combined diagnostic ROC curve showed no statistically significant differences in AUC values. The AUC values of these three curves were significantly higher than that of the triple iodine density diagnostic ROC curve, with all differences being statistically significant (*P* < 0.001) (see Figure 7 and Table 8 for details).

**TABLE 8:** Differences in AUC values among combined diagnostic ROC curves.

Project	AUC value difference	95%CI	*Z*	*P*
A4-A5	0.004	−0.005∼0.014	0.897	0.370
A4-B4	0.161	0.068∼0.255	3.374	0.001
A4-C	−0.005	222−0.018∼0.008	−0.796	0.426
A5-B4	0.165	0.074∼0.257	3.535	<0.001
A5-C	−0.001	−0.008∼0.006	−0.270	0.787
B4-C	−0.166	−0.255 to −0.078	−3.668	<0.001

## 4 Discussion

### 4.1 Clinical significance of demographic data in post-COVID syndrome

Analysis of general demographic data revealed no statistically significant differences in gender distribution between the groups, suggesting that pulmonary manifestations associated with post-COVID syndrome were independent of sex. In contrast, a significant age disparity was observed: the median age of the positive group was 74.5 years (IQR: 65.3–80.0), compared with 55.0 years (IQR: 44.8–63.0) in the negative group. Patients presenting positive clinical signs were generally older than those without, and this difference reached statistical significance.

D-dimer levels also differed markedly between the groups, with median values of 533.0 μg/L (IQR: 490.8–703.3) in the positive group versus 269.0 μg/L (IQR: 222.3–309.0) in the negative group. Patients exhibiting positive signs had significantly elevated D-dimer levels compared to those with negative signs. These findings were consistent with international literature and case reports, which suggest that after controlling for confounding factors, pulmonary lesions in post-COVID-19 syndrome correlate strongly with both age and D-dimer levels (8–10). Specifically, older individuals or those with higher D-dimer levels were more likely to manifest positive clinical signs.

Within the positive group, the likelihood of abnormal D-dimer values increased progressively with higher mMRC scores. Notably, no patient in the positive group exceeded an mMRC grade of 3, indicating that severe clinical symptoms were absent in this cohort. This observation aligned with existing literature suggesting that the clinical severity of post-COVID-19 syndrome may depend on the virulence of the infecting viral strain. Individuals previously infected with the Alpha or Delta variants are more prone to develop post-COVID-19 syndrome and tended to experience poorer prognoses and more severe sequelae compared to those infected with the Omicron variant (11–13).

In the present study, patients with the most severe mMRC score of 3 represented only 10.9% of the total sample and approximately 23.8% of those with positive physical signs. These patients were predominantly elderly, aged 70 years or older, further supporting the conclusion that among individuals with a history of Omicron infection, the risk of developing more pronounced clinical symptoms increases with advancing age.

### 4.2 Advantages of dual-energy CT perfusion over plain chest CT and CTPA

Tarraso et al. have investigated patients with post-COVID syndrome following infection with highly virulent strains and reported that plain chest CT can only detect anatomical abnormalities such as ground-glass opacities, solid nodular shadows, and sparse fine fibrous strands (18–21). These findings, while indicative of structural changes, do not significantly affect pulmonary V/Q and are insufficient to differentiate patients with post-COVID syndrome from control individuals without related clinical signs.

In the present study, plain chest CT scans revealed that, aside from a small subset of cases presenting abnormal density shadows, including ground-glass opacities, solid nodular lesions, sparse fine fibrous strands, and calcifications, no other significant parenchymal or mediastinal abnormalities were observed in either group. Similarly, CTPA images demonstrated well-filled pulmonary artery trunks and branches without evidence of embolism in both groups. Quantitative and qualitative analyses of these morphological parameters indicated no statistically significant differences between the positive and negative groups on either plain chest CT or CTPA.

In contrast, Mohamed et al. have highlighted that DECT perfusion imaging can detect subtle pulmonary perfusion abnormalities that are otherwise undetectable on standard chest CT (22). Consistent with these observations, DECT perfusion in the current study revealed variable degrees of hypoperfusion in the positive group, with some regions exhibiting complete perfusion defects. Compensatory hyperperfusion was frequently observed in contralateral lobes or other segments within the affected lobe. Lung perfusion volume-rendered (VR) images mirrored the DECT perfusion findings, presenting perfusion abnormalities in a three-dimensional format. Areas of reduced or absent perfusion appeared as deep yellow with sparse perfusion, whereas hyperperfused regions appeared as bright yellow with increased perfusion. Notably, neither DECT perfusion nor VR images in the negative group demonstrated significant perfusion abnormalities.

#### 4.3 Clinical value of dual-energy CT perfusion parameters in post-COVID-19 syndrome

Analysis of the measurement and calculation data revealed no statistically significant differences between the two groups in total lung volume or mean iodine density of normally perfused lung parenchyma. However, the absolute volume of normally perfused lung parenchyma in symptomatic patients, 2,920.7 cm^3^ (IQR: 2,736.1–3,104.4), was smaller than that in asymptomatic patients, 3,061.2 cm^3^ (IQR: 2,888.8–3,286.6). Previous studies indicate that in patients recovering from severe COVID-19 or those exhibiting more pronounced clinical symptoms, perfusion images often demonstrate bilateral perfusion defects, with the volume of hypoperfused or deficient lung tissue significantly exceeding that observed in patients with mild infections or without severe disease (15, 23).

Consistent with these reports, the present study found that the reduced/deficient lung volume in perfusion images of symptomatic patients, 1,44.9 cm^3^ (IQR: 96.5–197.0), was markedly greater than that in asymptomatic patients, 51.8 cm^3^ (IQR: 32.8–59.6). Furthermore, the mean iodine density in hypoperfused/deficient regions was lower in symptomatic patients, 0.2 mg/mL (IQR: 0.1–0.3), compared to 0.3 mg/mL (IQR: 0.2–0.5) in asymptomatic patients.

In addition, the volume of hyperperfused lung parenchyma in symptomatic patients, 88.4 cm^3^ (IQR: 56.0–109.3), exceeded that in asymptomatic patients, 39.8 cm^3^ (IQR: 35.5–43.8). However, the mean iodine density within hyperperfused regions was lower in symptomatic patients, 2.3 mg/mL (IQR: 2.2–2.5), compared with 2.6 mg/mL (IQR: 2.5–2.7) in asymptomatic patients. These findings might reflect microthrombus formation in regions of reduced or absent perfusion due to COVID-19-induced vascular endothelial injury, which leads to compensatory perfusion enhancement in the remaining lung tissue (6, 7, 24, 25).

Recent studies also suggest that the development of post-COVID-19 syndrome is associated with dysregulated T-lymphocyte-mediated immune responses, triggering localized immune-inflammatory reactions in affected tissues and organs (26). Pathologically, this manifests as inflammatory hyperemia within the capillary networks of these tissues, explaining why the volume of hyperperfused lung parenchyma is generally larger in symptomatic patients than in asymptomatic patients. The relative volume percentages of perfusion areas in both groups further corroborated these observations, supporting the functional significance of DECT perfusion parameters in identifying subtle pulmonary abnormalities in post-COVID-19 syndrome.

### 4.4 Diagnostic performance of dual-energy CT perfusion parameters

Bogot et al. investigated the relationship between pulmonary perfusion and ventilated lung in COVID-19 patients and healthy controls, finding a significant correlation between the volume of perfusion-abnormal regions and disease severity (27, 28). In the volume ROC curve analysis of this study, the AUC values for the absolute volume ROC curve of hypoperfused/infarct lung parenchyma, the dual diagnostic ROC curve of hypoperfused/infarct-hyperperfused lung parenchyma, and the triple volume diagnostic ROC curve all exceeded 0.9. The differences among these three curves were not statistically significant, suggesting comparable diagnostic efficacy and excellent performance for all three approaches. The AUC values for the enhanced parenchymal volume ROC curve and the normal parenchymal absolute volume ROC curve were 0.847 and 0.706, respectively. with significant statistical differences between these two AUC values and between them and the AUC values of the other three ROC curves. The AUC value of the perfusion-enhanced parenchymal volume ROC curve falls between 0.8 and 0.9, indicating good diagnostic performance. However, the AUC value of the normal parenchymal absolute volume ROC curve is 0.706, reflecting average diagnostic performance that is significantly inferior to the other four volume-based ROC curves.

Foti et al. observed in hospitalized patients that all included COVID-19 patients exhibited similar perfusion enhancement patterns in normally ventilated lung regions outside the lesion area, regardless of prognosis (29), indicating no significant iodine density differences. In the iodine density ROC curves of this study, the AUC values for the average iodine density of lung parenchyma in areas with reduced/deficient perfusion and those with enhanced perfusion were 0.738 and 0.767, respectively, falling within the range of 0.6 to 0.8. indicating moderate diagnostic performance. The AUC for the mean iodine density in parenchymal areas with normal perfusion was only 0.595 ( < 0.6), demonstrating poor diagnostic efficacy with considerable randomness. This finding aligns with conclusions from previous studies. However, combining all three parameters yielded an AUC of 0.823 for the ROC diagnostic curve, falling within the 0.8–0.9 range and achieving superior diagnostic performance.

Furthermore, some scholars have demonstrated that combining the volume of abnormal perfusion areas with the mean iodine density of these regions significantly improves the detection rate of pulmonary lesions caused by SARS-CoV-2 infection (30, 31). In this single-center, cross-sectional study, the combined diagnostic model demonstrated excellent diagnostic performance (AUC = 0.969) within our cohort.

In summary, in comparison with infections caused by the original Alpha variant and the Delta variant (32, 33), post-COVID-19 syndrome following Omicron infection generally manifests with milder clinical severity. This trend is likely attributable to the substantially reduced virulence of the Omicron variant as well as the widespread uptake of vaccination (34, 35).

### 4.5 Limitations of study

The primary limitations of this study include its relatively small sample size, single-center design, and the predominantly mild clinical presentation observed among most Omicron-infected patients with post-COVID-19 syndrome. In addition, many patients in China exhibit limited subjective awareness of post-COVID symptoms and variable compliance, posing challenges for large-scale, systematic investigations. The application of stringent inclusion and exclusion criteria further resulted in the omission of certain patients with underlying chronic conditions, as these comorbidities may also manifest with positive imaging and physical examination findings, complicating accurate diagnosis. Moreover, long-term investigations into the sequelae of COVID-19 remain limited globally, with research interest waning over time. The concurrent circulation of influenza A virus and human metapneumovirus, which can produce similar clinical symptoms and pulmonary perfusion patterns, introduces additional complexity and uncertainty to ongoing studies.

## 5 Conclusion

In summary, the “one-stop” pulmonary perfusion imaging achieved through the Lung Analysis module in the post-processing system, utilizing dual-energy CT with low-flow, low-dose CTPA technology, enhances patient safety while reducing the administration of iodinated contrast agents.

This technique accurately identifies functional abnormalities in lung parenchyma—difficult to detect via plain CT or conventional CTPA—in patients with post-COVID syndrome who previously contracted the Omicron variant. Compared to asymptomatic Omicron carriers, these patients primarily exhibit varying degrees of perfusion abnormalities in the lung parenchyma, (i.e., perfusion defects/reduction in some lung parenchymal areas and compensatory perfusion enhancement). The majority of patients exhibit relatively small volumes of perfusion abnormalities with mild severity.

Combining the volume of parenchymal areas with perfusion abnormalities and perfusion parameters such as mean iodine density with clinical data including mMRC grade and D-dimer levels significantly improves the accuracy of diagnosing pulmonary lesions in patients with post-COVID-19 syndrome. This shows improved discriminatory performance within the studied cohort and may support clinical assessment.

## Data Availability

The original contributions presented in the study are included in the article/supplementary material, further inquiries can be directed to the corresponding author.

## References

[1] Vink M Vink-Niese A. Could cognitive behavioural therapy be an effective treatment for long COVID and post COVID-19 fatigue syndrome? lessons from the qure study for Q-fever fatigue syndrome. *Healthcare.* (2020) 8:552. 10.3390/healthcare8040552 33322316 PMC7764131

[2] Huang C Huang L Wang Y Li X Ren L Gu X 6-month consequences of COVID-19 in patients discharged from hospital: a cohort study. *Lancet.* (2021) 401:e0021-33. 10.1016/S0140-6736(23)00810-3 37321233 PMC10258565

[3] El Sayed S Shokry D Gomaa SM. Post-COVID-19 fatigue and anhedonia: a cross-sectional study and their correlation to post-recovery period. *Neuropsychopharmacol Rep.* (2021) 41:50–5. 10.1002/npr2.12154 33332756 PMC8182964

[4] WHO *Coronavirus Disease (COVID-19): Post COVID-19 Condition.* Geneva: WHO (2023).

[5] Greenhalgh T Knight M A’Court C Buxton M Husain L. Management of post-acute covid-19 in primary care. *BMJ.* (2020) 370:m3026. 10.1136/bmj.m3026 32784198

[6] Jimeno-Almazán A Pallarés JG Buendía-Romero Á Martínez-Cava A Franco-López F Sánchez-Alcaraz Martínez BJ Post-COVID-19 syndrome and the potential benefits of exercise. *Int J Environ Res Public Health.* (2021) 18:5329. 10.3390/ijerph18105329 34067776 PMC8156194

[7] Lee JH Koh J Jeon YK Goo JM Yoon SH. An integrated radiologic-pathologic understanding of COVID-19 pneumonia. *Radiology.* (2023) 306:e222600. 10.1148/radiol.222600 36648343 PMC9868683

[8] Amir M Ali U Javed A Ahmad J. Utility of inflammatory markers for tocilizumab in covid-19 patients: a single-site retrospective study. *J Ayub Med Coll Abbottabad.* (2022) 34:777–81. 10.55519/JAMC-04-10565 36566398

[9] Farid E Sridharan K Alsegai OA Khawaja SA Mansoor EJ Teraifi NA Utility of inflammatory biomarkers in patients with COVID-19 infections: bahrain experience. *Biomark Med.* (2021) 15:541–9. 10.2217/bmm-2020-0422 33988463 PMC8120999

[10] Jůza T Válek V Vlk D Dostál M Andrašina T. Roles of spectral dual-layer CT, D-dimer concentration, and COVID-19 pneumonia in diagnosis of pulmonary embolism. *Eur J Radiol Open.* (2024) 12:100575. 10.1016/j.ejro.2024.100575 38882633 PMC11179566

[11] Hattab D Amer MFA Al-Alami ZM Bakhtiar A. SARS-CoV-2 journey: from alpha variant to omicron and its sub-variants. *Infection.* (2024) 52:767–86. 10.1007/s15010-024-02223-y 38554253 PMC11143066

[12] Miller NL Clark T Raman R Sasisekharan R. Insights on the mutational landscape of the SARS-CoV-2 Omicron variant receptor-binding domain. *Cell Rep Med.* (2022) 3:100527. 10.1016/j.xcrm.2022.100527 35233548 PMC8784435

[13] Alizon S Haim-Boukobza S Foulongne V Verdurme L Trombert-Paolantoni S Lecorche E Rapid spread of the SARS-CoV-2 Delta variant in some French regions. *June 2021. Euro Surveill.* (2021) 26:2100573. 10.2807/1560-7917.ES.2021.26.28.2100573 34269174 PMC8284044

[14] Michishita T Saji R Miyazaki H Mishima S Shimada K Minami S Utility of dual-energy computed tomography in the association of COVID-19 pneumonia severity. *Acute Med Surg.* (2022) 9:e811. 10.1002/ams2.811 36570597 PMC9767859

[15] Aydın S Karavaş E Ünver E Şenbil DC Kantarcı M. Long-term lung perfusion changes related to COVID-19: a dual energy computed tomography study. *Diagn Interv Radiol.* (2023) 29:103–8. 10.5152/dir.2022.211090 36960546 PMC10679602

[16] Farkas B Képes Z Barna SK Szugyiczki V Bakos M Forgács A Unusual perfusion patterns on perfusion-only SPECT/CT scans in COVID-19 patients. *Ann Nucl Med.* (2022) 36:804–11. 10.1007/s12149-022-01761-5 35763163 PMC9244068

[17] Matheson AM McIntosh MJ Kooner HK Lee J Desaigoudar V Bier E Persistent 129Xe MRI pulmonary and CT vascular abnormalities in symptomatic individuals with post-acute COVID-19 syndrome. *Radiology.* (2022) 305:466–76. 10.1148/radiol.220492 35762891 PMC9272782

[18] Tarraso J Safont B Carbonell-Asins JA Fernandez-Fabrellas E Sancho-Chust JN Naval E Lung function and radiological findings 1 year after COVID-19: a prospective follow-up. *Respir Res.* (2022) 23:242. 10.1186/s12931-022-02166-8 36096801 PMC9466319

[19] Wu X Liu X Zhou Y Yu H Li R Zhan Q 3-month, 6-month, 9-month, and 12-month respiratory outcomes in patients following COVID-19-related hospitalisation: a prospective study. *Lancet Respir Med.* (2021) 9:747–54. 10.1016/S2213-2600(21)00174-0 33964245 PMC8099316

[20] Huang L Yao Q Gu X Wang Q Ren L Wang Y 1-year outcomes in hospital survivors with COVID-19: a longitudinal cohort study. *Lancet.* (2021) 398:747–58. 10.1016/S0140-6736(21)01755-4 34454673 PMC8389999

[21] Schlemmer F Valentin S Boyer L Guillaumot A Chabot F Dupin C Respiratory recovery trajectories after severe-to-critical COVID-19: a 1-year prospective multicentre study. *Eur Respir J.* (2023) 61:2201532. 10.1183/13993003.01532-2022 36669777 PMC10066566

[22] Mohamed I de Broucker V Duhamel A Giordano J Ego A Fonne N Pulmonary circulation abnormalities in post-acute COVID-19 syndrome: dual-energy CT angiographic findings in 79 patients. *Eur Radiol.* (2023) 33:4700–12. 10.1007/s00330-023-09618-9 37145145 PMC10129318

[23] Price LC Garfield B Bloom C Jeyin N Nissan D Hull JH Persistent isolated impairment of gas transfer following COVID-19 pneumonitis relates to perfusion defects on dual-energy computed tomography. *ERJ Open Res.* (2022) 8:224–2022. 10.1183/23120541.00224-2022 36447736 PMC9548240

[24] Ali L Sharif M Naqvi SGA Mohammed I Baig MA Sidratul Muntaha K To study the correlation of clinical severity and cytokine storm in COVID-19 pulmonary embolism patients by using Computed Tomography Pulmonary Angiography (CTPA) qanadli clot burden scoring system. *Cureus.* (2023) 15:e39263. 10.7759/cureus.39263 37342749 PMC10278873

[25] Ackermann M Verleden SE Kuehnel M Haverich A Welte T Laenger F Pulmonary vascular endothelialitis, thrombosis, and angiogenesis in Covid-19. *N Engl J Med.* (2020) 383:120–8. 10.1056/NEJMoa2015432 32437596 PMC7412750

[26] Lo PC Feng JY Hsiao YH Su KC Chou KT Chen YM Long COVID symptoms after 8-month recovery: persistent static lung hyperinflation associated with small airway dysfunction. *Respir Res.* (2024) 25:209. 10.1186/s12931-024-02830-1 38750527 PMC11097537

[27] Bogot NR Steiner R Helviz Y Weiss C Cherniavsky K Pichkhadze O Distribution of aeration and pulmonary blood volume in healthy, ARDS and COVID-19 lungs: a dual-energy computed tomography retrospective cohort study. *Acad Radiol.* (2023) 30:2548–56. 10.1016/j.acra.2023.01.016 36966073 PMC10035816

[28] Ball L Robba C Herrmann J Gerard SE Xin Y Mandelli M Lung distribution of gas and blood volume in critically ill COVID-19 patients: a quantitative dual-energy computed tomography study. *Crit Care.* (2021) 25:214. 10.1186/s13054-021-03610-9 34154635 PMC8215486

[29] Foti G Longo C Faccioli N Guerriero M Stefanini F Buonfrate D. Quantitative assessment of lung volumes and enhancement in patients with COVID-19: role of Dual-Energy CT. *Diagnostics.* (2023) 13:1201. 10.3390/diagnostics13061201 36980509 PMC10047841

[30] Grillet F Busse-Coté A Calame P Behr J Delabrousse E Aubry S. COVID-19 pneumonia: microvascular disease revealed on pulmonary dual-energy computed tomography angiography. *Quant Imaging Med Surg.* (2020) 10:1852–62. 10.21037/qims-20-708 32879862 PMC7417764

[31] Remy-Jardin M Duthoit L Perez T Felloni P Faivre JB Fry S Assessment of pulmonary arterial circulation 3 months after hospitalization for SARS-CoV-2 pneumonia: dual-energy CT (DECT) angiographic study in 55 patients. *EClinicalMedicine.* (2021) 34:100778. 10.1016/j.eclinm.2021.100778 33817609 PMC8008988

[32] Yang N Wang C Huang J Dong J Ye J Fu Y Clinical and pulmonary CT characteristics of patients infected with the SARS-CoV-2 Omicron variant compared with those of patients infected with the alpha viral strain. *Front Public Health.* (2022) 10:931480. 10.3389/fpubh.2022.931480 35903393 PMC9315283

[33] Tsakok MT Watson RA Saujani SJ Kong M Xie C Peschl H Reduction in chest CT severity and improved hospital outcomes in SARS-CoV-2 Omicron compared with delta variant infection. *Radiology.* (2023) 306:261–9. 10.1148/radiol.220533 35727150 PMC9272784

[34] Antonelli M Pujol JC Spector TD Ourselin S Steves CJ. Risk of long COVID associated with delta versus omicron variants of SARS-CoV-2. *Lancet.* (2022) 399:2263–4. 10.1016/S0140-6736(22)00941-2 35717982 PMC9212672

[35] Lee JE Hwang M Kim YH Chung MJ Sim BH Jeong WG SARS-CoV-2 variants infection in relationship to imaging-based pneumonia and clinical outcomes. *Radiology.* (2023) 306:e221795. 10.1148/radiol.221795 36165791 PMC9527969

